# Supplementary material for: Tracking miR-17-5p Levels following Expression of Seven Reported Target mRNAs
Source: Cancers (Basel). 2022 May 24;14(11):2585. doi: 10.3390/cancers14112585 (PMC9179866; doi:10.3390/cancers14112585)
Supplement: Supplementary file 1 [file cancers-14-02585-s001.zip › cancers-1610296-supplementary.pdf]

Figure S1. Primer Sequences.

Primer Name	Sequence	Source / Repository
hsa-miR-17-5p	5′ caaagtgcttacagtgcagg	Eurofins Genomics LLC
hsa-miR-17-3p	5′ caaagtgcttacagtgcagg	Eurofins Genomics LLC
hsa-miR-24	5′ tgcctactgagctgatatca	Eurofins Genomics LLC
hsa-miR-93	5′ caaagtgctgttcgtgcagg	Eurofins Genomics LLC
hsa-miR-98	5′ tgaggtagtaagttgtattg	Eurofins Genomics LLC
hsa-miR199a-3p	5′ acagtagtctgcacattggt	Eurofins Genomics LLC
hsa-miR-299-3p	5′ tatgtgggatggtaaaccgc	Eurofins Genomics LLC
hsa-miR-330-3p	5′ gcaaagcacacggcctgcag	Eurofins Genomics LLC
hsa-miR-378	5′ ctcctgactccaggtcctgt	Eurofins Genomics LLC
hsa-miR491-5p	5′ agtggggaacccttccatga	Eurofins Genomics LLC
hsa-miR-661	5′ tgcctgggtctctggcctgc	Eurofins Genomics LLC
hu.PTEN-UTR-F,	5′ tgaattttttttatcaagagggat	Eurofins Genomics LLC
hu.PTEN-UTR-R	5′ gtg caaa gggg tagg at gtg aac	Eurofins Genomics LLC
hu.Timp2-UTR-F,	5′ ctgacatcccttcctggaaacagcatg	Eurofins Genomics LLC
hu.Timp2-UTR-R	5′ ccca gc tggg aga tccct aagtg ct	Eurofins Genomics LLC
hu.Timp3-UTR-F	5′ tgagcgccagaccctgccccacctcac	Eurofins Genomics LLC
hu.Timp3-UTR-R	5′ ga cctttc tttaaa tgtt cc aagt gc	Eurofins Genomics LLC
hu.ADCY5-UTR-F	5′ tagcagataccagccagcggtgcc	Eurofins Genomics LLC
hu.ADCY5-UTR-R	5′ ca gaag ttg cttct gag tcaa	Eurofins Genomics LLC
hu.IRS1-UTR-F	5′ acctaccttgtgtgttctgggaac	Eurofins Genomics LLC
hu.IRS1-UTR-R	5′ gttg at ca gggc aac tggg ca	Eurofins Genomics LLC
hu.PPAR-α-UTR-F	5′ tgatatgg agacactgtg tatggc	Eurofins Genomics LLC
hu.PPAR-α-UTR-R	5′ attt cca ta cgc ta cca gc at cccg	Eurofins Genomics LLC
hu.STAT3-UTR-F	5′ ggtgatga gtttccgggt gtctga	Eurofins Genomics LLC
hu.STAT3-UTR-R	5′ agg tgcc aggggg caaa gaga gaa	Eurofins Genomics LLC
hu.FN-UTR-F	5′ taaatcatctttccaatc	Eurofins Genomics LLC
hu.FN-UTR-R	5′ t gag ct gaa gc tgg ag aa	Eurofins Genomics LLC
mu.p21-F	5′ gtggaactttgacttcgtcacgga	Eurofins Genomics LLC
mu.p21-R	5′ agag tg caa ga ca gcg acaa ggcc	Eurofins Genomics LLC
mu.bcl2l11-F	5′ aattgcagcctgctgagaggcctc	Eurofins Genomics LLC
mu.bcl2l11-R	5′ acc ggg aca gc agag aaga tc ttc	Eurofins Genomics LLC
mu.RB1-F	5′ tcatctaatggacttccagaggtt	Eurofins Genomics LLC
mu.RB1-R	5′ cct aac tgg ag tgtgt ggag taac	Eurofins Genomics LLC
mu.cyclinD1-F	5′ gatgctggaggtctgtgaggagca	Eurofins Genomics LLC
mu.cyclinD1-R	5′ cgg ata gag ttgt ca gtgt aga tg	Eurofins Genomics LLC
mu.NCOA3-F	5′ agacttgcagtgctgtatgatctg	Eurofins Genomics LLC
mu.NCOA3-R	5′ ca tg ac tg ccca tc att ca ga ctg	Eurofins Genomics LLC
mu.PTEN-F	5′ cgctgcctcggctgccaggcctct	Eurofins Genomics LLC
mu.PTEN-R	5′ gag gagagaga tgg ca gaag ct gc	Eurofins Genomics LLC
mu.E2F1-F	5′ catccagctcattgccaagaagtc	Eurofins Genomics LLC
mu.E2F1-R	5′ aca ta ggcc agg cgc tggg tgt cg	Eurofins Genomics LLC
mu.Rbl2-F	5′ tgctccttacacgacggtctagtg	Eurofins Genomics LLC
mu.Rbl2-R	5′ tgctccttacacgacggtctagtg	Eurofins Genomics LLC
mu.PCAF-F	5′ tgaaccgcatcaactactggcatc	Eurofins Genomics LLC
mu.PCAF-R	5′ gt cg tctc at ga tggt gaaga ccg	Eurofins Genomics LLC
mu.CTGF-F	5′ catggcgtaaagccaggaagtaag	Eurofins Genomics LLC
mu.CTGF-R	5′ gacataacgttctcactttggtgg	Eurofins Genomics LLC
mu.STAT3-F	5′ ccgacccaggtagtgctgc	Eurofins Genomics LLC
mu.STAT3-R	5′ caatggtattgctgcaggtcg	Eurofins Genomics LLC
mu.ADCY5-F	5′ tagcagataccagccagcggtgcc	Eurofins Genomics LLC
mu.ADCY5-R	5′ ca gaag ttg cttct gag tcaa	Eurofins Genomics LLC
miR-17-genotyping F1	5′ccaccggtcgccaccatggtgagcaaggg	Eurofins Genomics LLC
miR-17-genotyping R1	5′gaag aaga tggt gcgc tcct gga cg tag	Eurofins Genomics LLC
miR-17-genotyping F2	5′ tccgctagcgctaccggactcagatct	Eurofins Genomics LLC
miR-17-genotyping R2	5′ ga cca ga tc ag tgg tctc ata ca gaag	Eurofins Genomics LLC

Figure S2. Potential hsa-miR-17-5p (5′ CAAAGUGCUUACAGUGCAGGUAG**)** **target sequences** (target site in blue, mutation site in red).

p21 (*CDKN1A*) (a),
acagatg**gca ctttg**aaggg
gttcatt**gca cttt**gattag

bcl2l11 (c),
taga**gcactt ta**ctctgttt
ggtaattgc**c acttta**cttg

E2F1 (d),
ctaact**gcac ttt**cggccct
ctccaatct**g cacttt**gatt

Rb1 (e),
tatatcccaa gt**gcacttt**c taatgtttct

Rbl2 (f),
gag**gcacttt a**gggctgtaa
gata**gcactt t**ctacaatgt

cyclin D1 (g),
gt**cacttta**t aagtcattgt
caa**gcacttt** cagtccaata

NCOA3 (h),
tgtgcagctg agt**gcacttt a**tttaaaaag
actaa**gcact tt**gttaattt gggggggaaag

PCAF (KAT2B) (i),
taataatta**g cacttt**tgaa aaaacaaaaa

CTGF (j),
gaaatgtggt agcct**cactt t**taatgaaca

PTEN (b),
atg**gcacttt** cccgttttat
attaataaag atg**cgtgaaa** cccgttttat

STAT3 (k),
g**gcacttt**ta aaaatagaga
g**gcagaaa**ta aaaatagaga

ADCY5 (l).
tgctggtaaa attcccactt tgactcagaa
tgctggtaaa attccgtgaaacactcagaa

FN,
5' taatgttgcattaga**gcactttg**caattgc
5' taatgttgcattaga**ggtgaaac**caattgc

TIMP3,

5' tttcttgcaaatttagcacttggaacatttaaa

5′ tttcttgcaaatttagctgaaggaacatttaaa

TIMP2,

5′ ccaagcaggcagcacttagggatctcc

5′ ccaagcaggcaggtgaaagggatctcc

PPARa,

5′ aaaactaatctgcactttttaacctttgaaaa

5′ aaaactaatctgcagaatttaacctttgaaaa

Figure S3. 12 selected miR-17-5p target genes.

Gene Name	RefSeq (mRNA)	Function
**p21 (cyclin-dependent kinase inhibitor 1)**	NM_078467 (human)<br>NM_001111099 (mouse)	1.p21 is a potent cyclin-dependent kinase inhibitor (CKI).<br>2.p21 interacts with proliferating cell nuclear antigen (PCNA), a DNA polymerase accessory factor, and plays a regulatory role in S phase DNA replication and DNA damage repair.<br>3.This protein was reported to be specifically cleaved by CASP3-like caspases, which thus leads to a dramatic activation of CDK2, and may be instrumental in the execution of apoptosis following caspase activation.
**Phosphatase and tensin homolog (PTEN)**	NM_000314 (human)<br>NM_008960 (mouse)	1.PTEN protein acts as a phosphatase to dephosphorylate phosphatidylinositol (3,4,5)-trisphosphate (PtdIns (3,4,5)P3 or PIP3).<br>2.PTEN also has weak protein phosphatase activity, but this activity is also crucial for its role as a tumor suppressor.
**Bcl-2-like protein 11 (BCL2L11)**	NM_001204106 (human)<br>NM_001284410 (mouse)	BCL-2 family members form hetero- or homodimers and act as anti- or pro-apoptotic regulators that are involved in a wide variety of cellular activities.
**Transcription factor E2F1 (E2F1)**	NM_005225 (human)<br>NM_007891 (mouse)	The E2F family plays a crucial role in the control of cell cycle and action of tumor suppressor proteins and is also a target of the transforming proteins of small DNA tumor viruses.
**Retinoblastoma protein (Rb1)**	NM_000321 (human)<br>NM_009029 (mouse)	RB1 play roles in regulating cell cycle, and it induce cell senescence.
**Retinoblastoma-like protein 2 (Rbl2)**	NM_005611 (human)<br>NM_001282000 (mouse)	1.Key regulator of entry into cell division.<br>2.Directly involved in heterochromatin formation by maintaining overall chromatin structure and, in particular, that of constitutive heterochromatin by stabilizing histone methylation.
**Cyclin D1**	NM_053056 (human)<br>NM_007631 (mouse)	Cyclins function as regulators of CDKs (Cyclin-dependent kinase). Different cyclins exhibit distinct expression and degradation patterns which contribute to the temporal coordination of each mitotic event. This cyclin forms a complex with and functions as a regulatory subunit of CDK4 or CDK6, whose activity is required for cell cycle G1/S transition. This protein has been shown to interact with tumor suppressor protein Rb and the expression of this gene is regulated positively by Rb. Mutations, amplification and overexpression of this gene, which alters cell cycle progression, are observed frequently in a variety of tumors and may contribute to tumorigenesis.
**Tumor protein p53-inducible nuclear protein 1 (TP53INP1)**	NM_001135733 (human)<br>NM_001199105 (mouse)	TP53INP1 functions as a Tumor Suppressor and induces apoptosis through phosphorylating p53 at Serine-46. Multiple lines of evidence suggest that TP53INP1 gene expression is modulated by p53.
**P300/CBP-associated factor (PCAF)**	NM_003884 (human)<br>NM_001190846 (mouse)	CBP and p300 are large nuclear proteins that bind to many sequence-specific factors involved in cell growth and/or differentiation, including c-jun and the adenoviral oncoprotein E1A. The protein encoded by the PCAF gene associates with p300/CBP. It has *in vitro* and *in vivo* binding activity with CBP and p300, and competes with E1A for binding sites in p300/CBP. It has histone acetyl transferase activity with core histones and nucleosome core particles, indicating that this protein plays a direct role in transcriptional regulation.
**Connective tissue growth factor (CTGF)**	NM_001901 (human)<br>NM_010217 (mouse)	CTGF has important roles in many biological processes, including cell adhesion, migration, proliferation, angiogenesis, skeletal development, and tissue wound repair, and is critically involved in fibrotic disease and several forms of cancers.
**Signal transducer and activator of transcription 3 (STAT3)**	NM_003150 (human)<br>NM_011486 (mouse)	1.STAT3 is a member of the STAT protein family. In response to cytokines and growth factors, STAT3 is phosphorylated by receptor-associated Janus kinases (JAK), forms homo- or heterodimers, and translocates to the cell nucleus where it acts as a transcription activator.<br>2.STAT3 mediates the expression of a variety of genes in response to cell stimuli, and thus plays a key role in many cellular processes such as cell growth and apoptosis
**Adenylyl cyclase type 5 (ADCY5)**	NM_001199642 (mouse)<br>NM_001012765 (mouse)	This enzyme helps convert a molecule called adenosine triphosphate (ATP) to another molecule called cyclic adenosine monophosphate (cAMP).

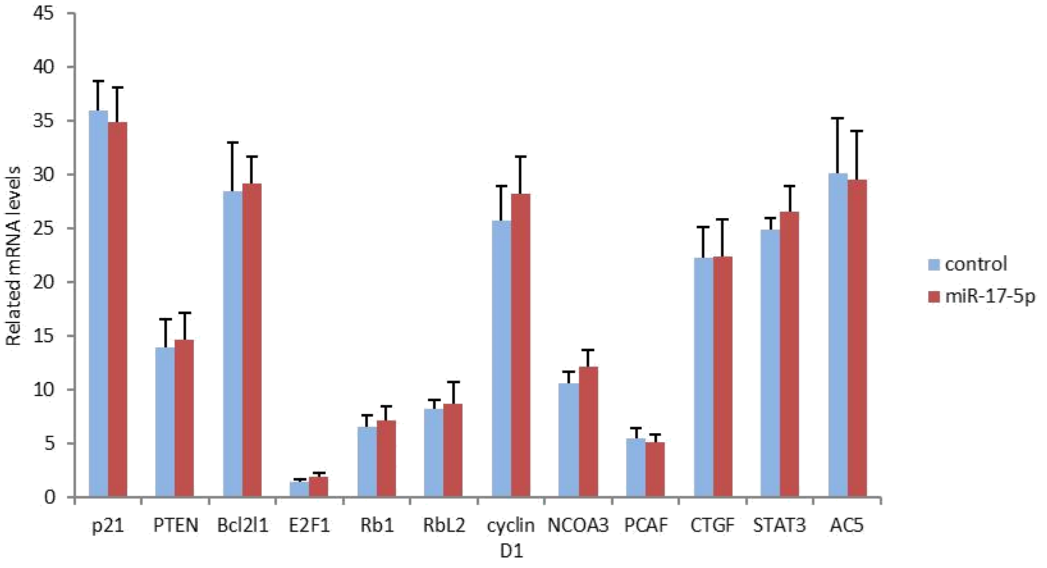

Figure S4. 4T1 cells were transfected with control oligo and miR-17-5p, and subjected to RT-PCR. Expression of miR-17-5p did not change p21, PTEN, bcl2l11, E2F1, Rb1, Rbl2, cyclin D1, NCOA3, PCAF, CTGF, STAT3, and ADCY5 expression at mRNA levels. *p<0.05, **p<0.01 vs control; Error bars, SD; n=5.

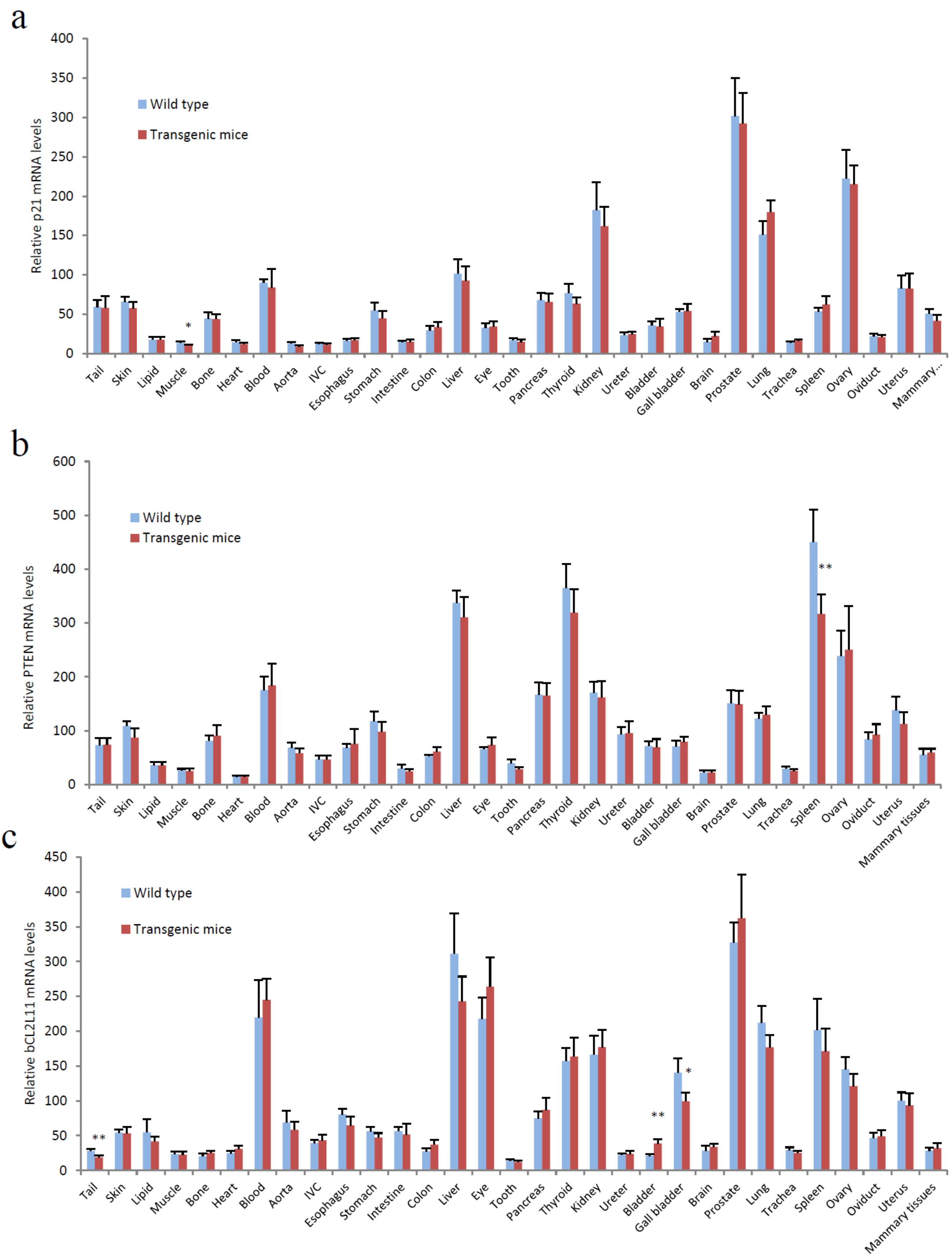
a
Relative p21 mRNA levels
Wild type
Transgenic mice
0
50
100
150
200
250
300
350
400
*
Tail Skin Lipid Muscle Bone Heart Blood Aorta IVC Esophagus Stomach Intestine Colon Liver Eye Tooth Pancreas Thyroid Kidney Ureter Bladder Gall bladder Brain Prostate Lung Trachea Spleen Ovary Oviduct Uterus Mammary...
b
Relative PTEN mRNA levels
Wild type
Transgenic mice
0
100
200
300
400
500
600
**
Tail Skin Lipid Muscle Bone Heart Blood Aorta IVC Esophagus Stomach Intestine Colon Liver Eye Tooth Pancreas Thyroid Kidney Ureter Bladder Gall bladder Brain Prostate Lung Trachea Spleen Ovary Oviduct Uterus Mammary tissues
c
Relative bCL2L11 mRNA levels
Wild type
Transgenic mice
0
50
100
150
200
250
300
350
400
450
**
**
*
Tail Skin Lipid Muscle Bone Heart Blood Aorta IVC Esophagus Stomach Intestine Colon Liver Eye Tooth Pancreas Thyroid Kidney Ureter Bladder Gall bladder Brain Prostate Lung Trachea Spleen Ovary Oviduct Uterus Mammary tissues

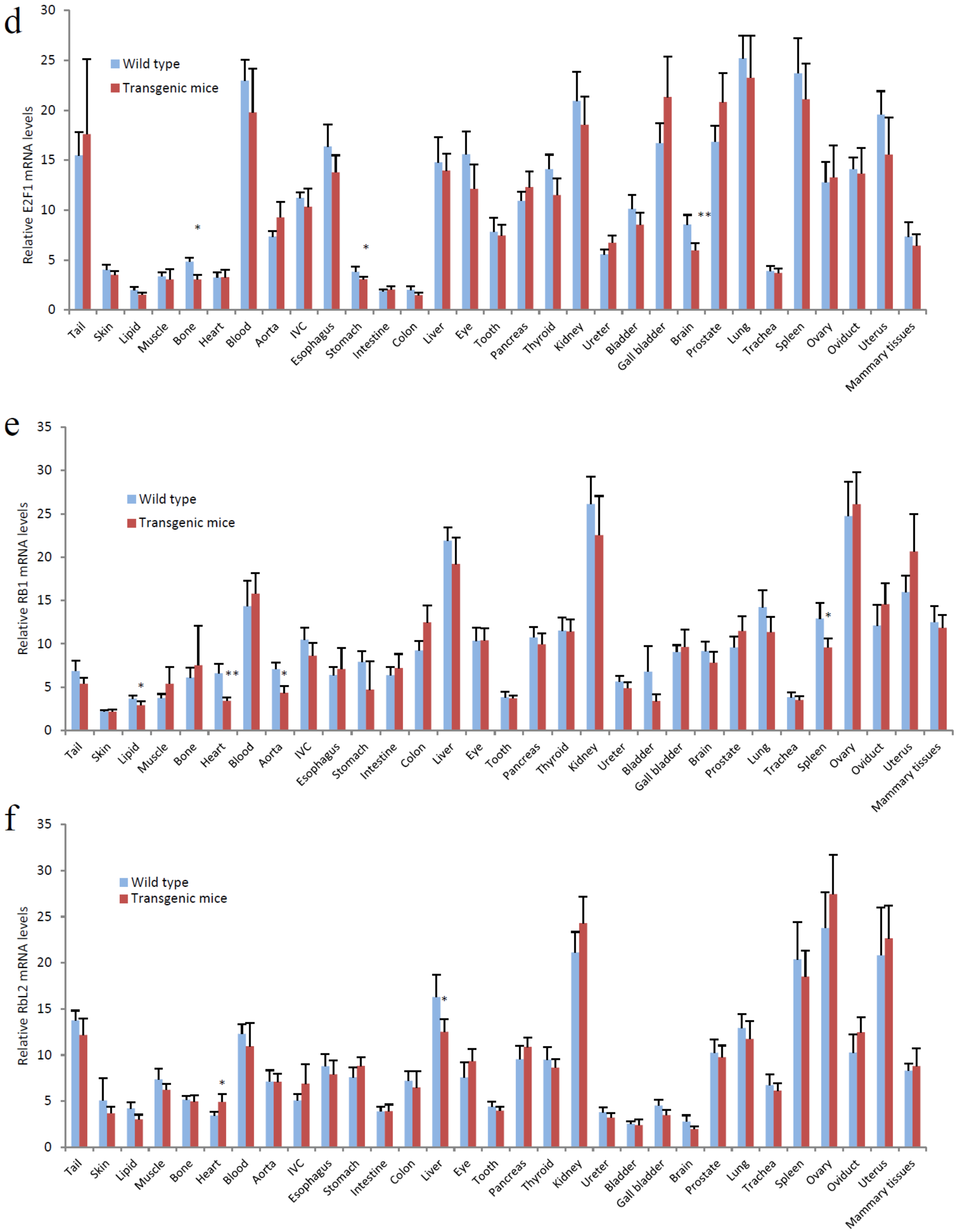
d
Wild type
Transgenic mice
Relative E2F1 mRNA levels
0
5
10
15
20
25
30
*
**
Tail
Skin
Lipid
Muscle
Bone
Heart
Blood
Aorta
IVC
Esophagus
Stomach
Intestine
Colon
Liver
Eye
Tooth
Pancreas
Thyroid
Kidney
Ureter
Bladder
Gall bladder
Brain
Prostate
Lung
Trachea
Spleen
Ovary
Oviduct
Uterus
Mammary tissues
e
Wild type
Transgenic mice
Relative RB1 mRNA levels
0
5
10
15
20
25
30
35
*
**
f
Wild type
Transgenic mice
Relative RbL2 mRNA levels
0
5
10
15
20
25
30
35
*

g

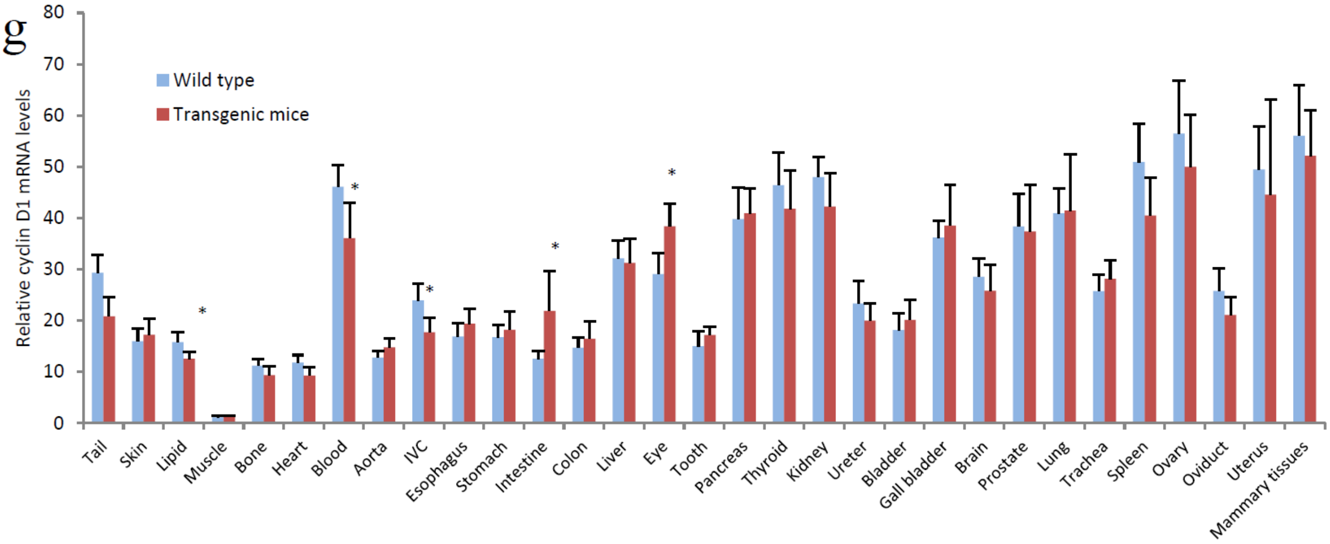

Wild type
Transgenic mice
Relative cyclin D1 mRNA levels
0
10
20
30
40
50
60
70
80
*
Tail
Skin
Lipid
Muscle
Bone
Heart
Blood
Aorta
IVC
Esophagus
Stomach
Intestine
Colon
Liver
Eye
Tooth
Pancreas
Thyroid
Kidney
Ureter
Bladder
Gall bladder
Brain
Prostate
Lung
Trachea
Spleen
Ovary
Oviduct
Uterus
Mammary tissues

h

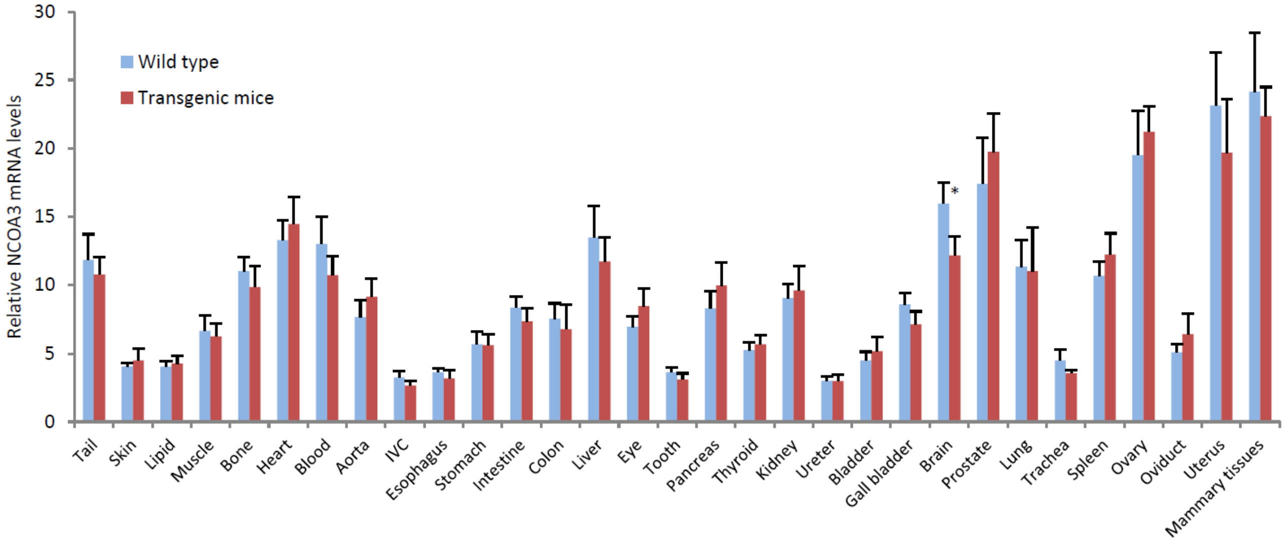

Wild type
Transgenic mice
Relative NCOA3 mRNA levels
0
5
10
15
20
25
30
*
Tail
Skin
Lipid
Muscle
Bone
Heart
Blood
Aorta
IVC
Esophagus
Stomach
Intestine
Colon
Liver
Eye
Tooth
Pancreas
Thyroid
Kidney
Ureter
Bladder
Gall bladder
Brain
Prostate
Lung
Trachea
Spleen
Ovary
Oviduct
Uterus
Mammary tissues

i

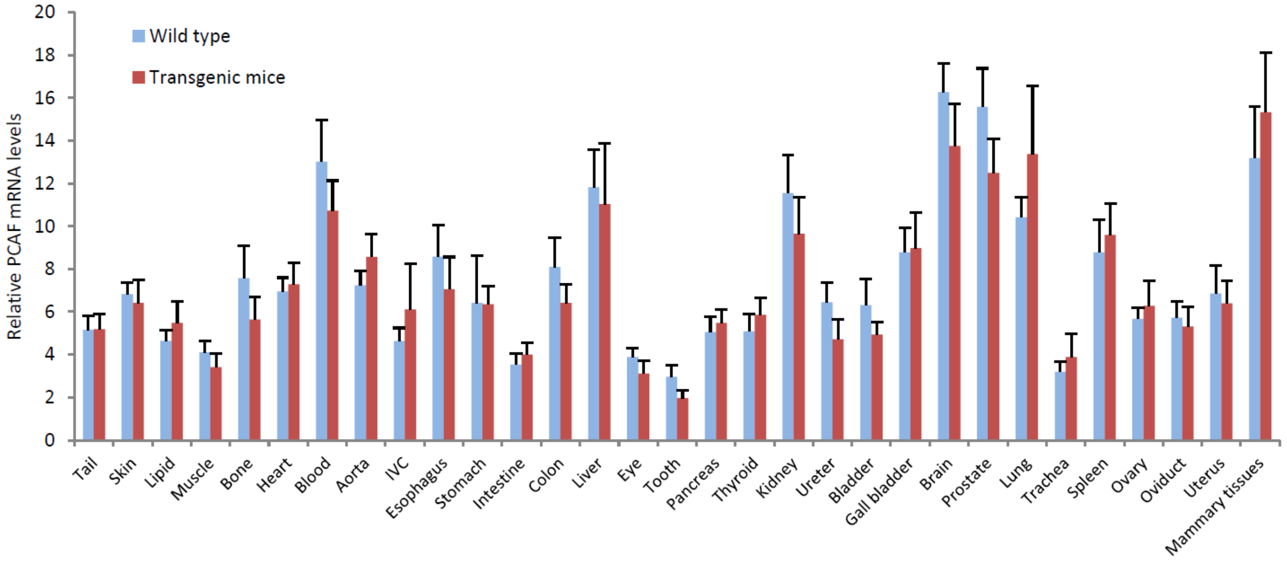

Wild type
Transgenic mice
Relative PCAF mRNA levels
0
2
4
6
8
10
12
14
16
18
20
Tail
Skin
Lipid
Muscle
Bone
Heart
Blood
Aorta
IVC
Esophagus
Stomach
Intestine
Colon
Liver
Eye
Tooth
Pancreas
Thyroid
Kidney
Ureter
Bladder
Gall bladder
Brain
Prostate
Lung
Trachea
Spleen
Ovary
Oviduct
Uterus
Mammary tissues

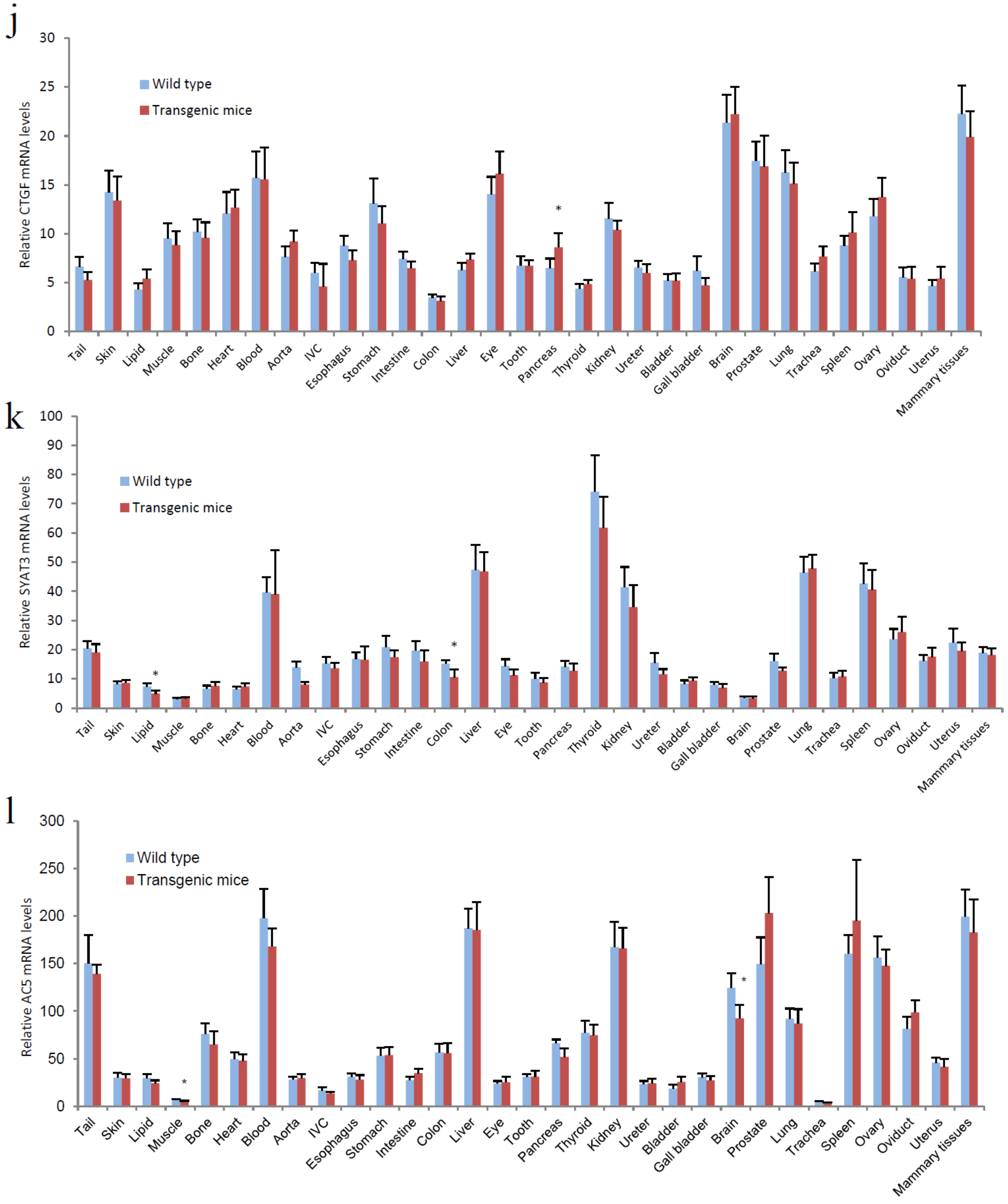

Figure S5. Real-time PCR using primers against 12 selected miR-17-5p target genes, including p21(a), PTEN (b), bcl2l11 (c), E2F1 (d), Rb1 (e), Rbl2 (f), cyclin D1 (g), NCOA3 (h), PCAF (i), CTGF (j), STAT3 (k), and ADCY5 (l). *p<0.05, **p<0.01 vs wt; Error bars, SD; n=4.

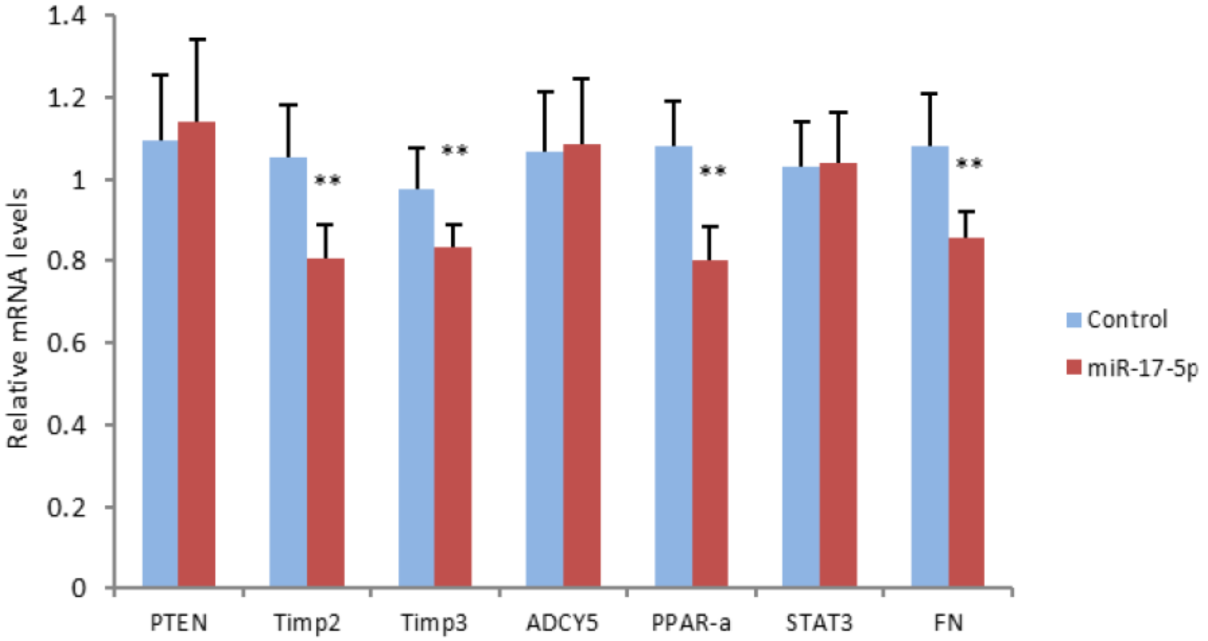

Figure S6. HepG2 cells were transfected with control oligo and miR-17-5p, and subjected to RT-PCR. Expression of miR-17-5p decreased Timp2, Timp3, PPAR-α, and Fibronectin (FN) expression on mRNA levels, but did not significantly change PTEN, ADCY5 or STAT3 mRNA levels. **p<0.01 vs control; Error bars, SD; n=5.

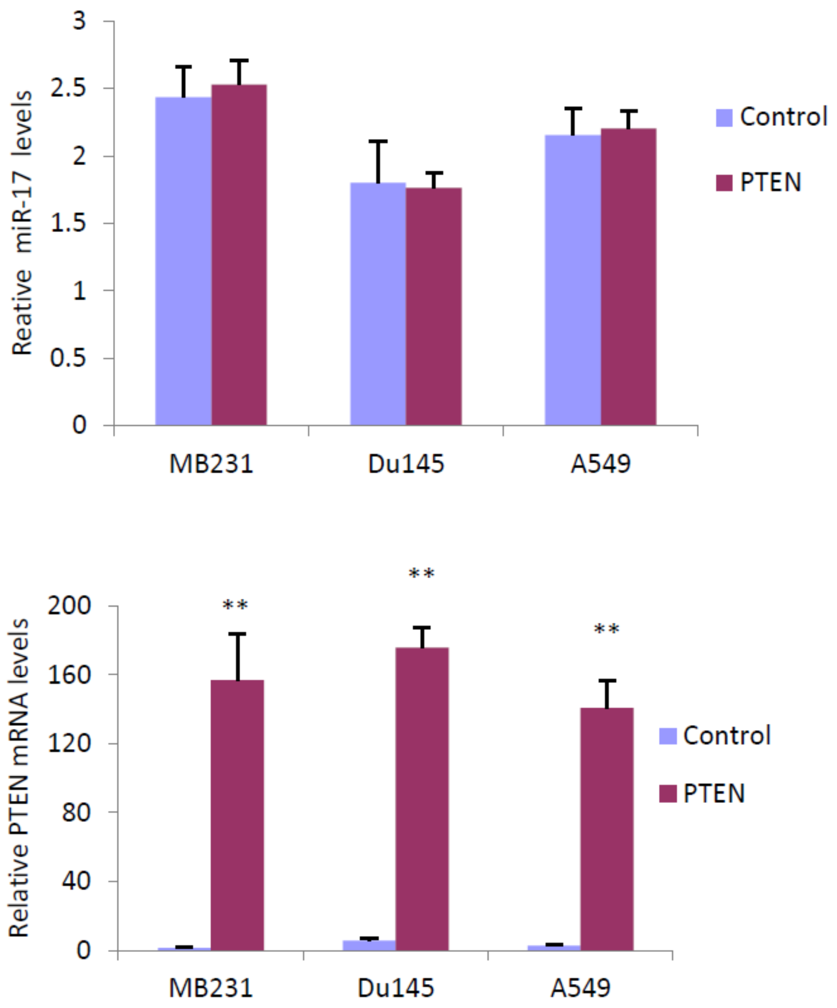

Figure S7. Upper, a control vector or PTEN coding sequence plasmid was transfected to breast cancer (MDA-MB-231), prostate cancer (DU145), and lung cancer cell lines (A549). The expression of PTEN coding sequence plasmid did not change miR-17-5p levels in all the cell lines used. Lower, expression of PTEN mRNA in the transfected cell lines. **p<0.01 vs control; Error bars, SD; n=5.

a

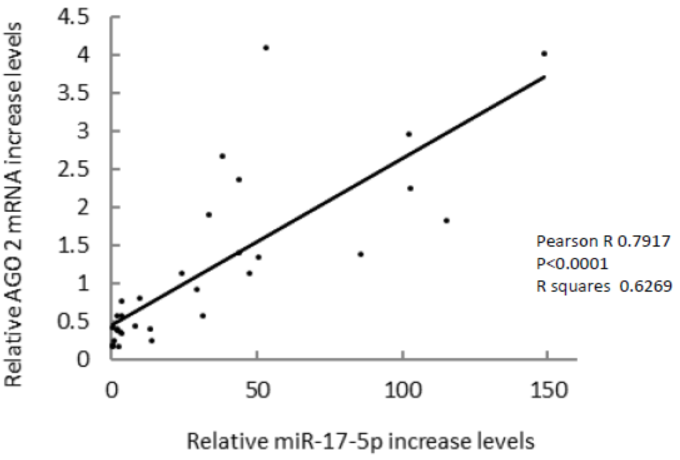

b

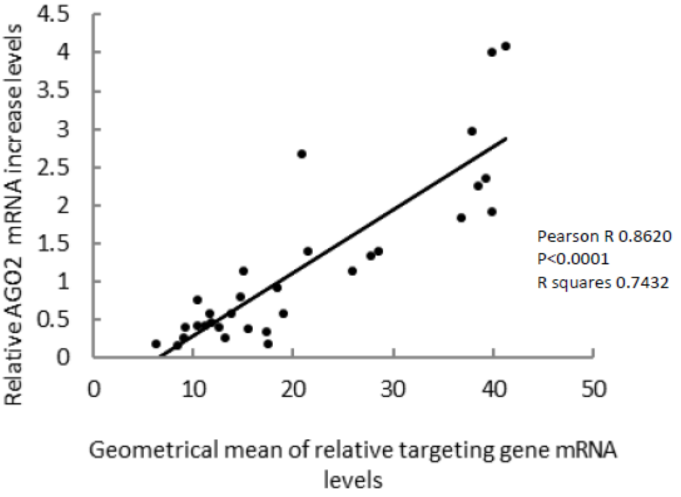

Figure S8. Pearson correlation (a). Pearson correlation between relative increases of AGO2 mRNA and miR-17-5p in organs of transgenic mice was analyzed by Prism 8. Pearson R=0.7917; p<0.0001; n=31. (b). Pearson correlation between relative increases of AGO2 mRNA and geometrical mean of mRNA levels of 12 miR-17-5p target genes in organs was analyzed by Prism 8. Fold AGO2 mRNA was positively correlated with geometrical mean of mRNA levels of these 12 miR-17-5p target genes. Pearson R=0.8620; p<0.0001; n=31.

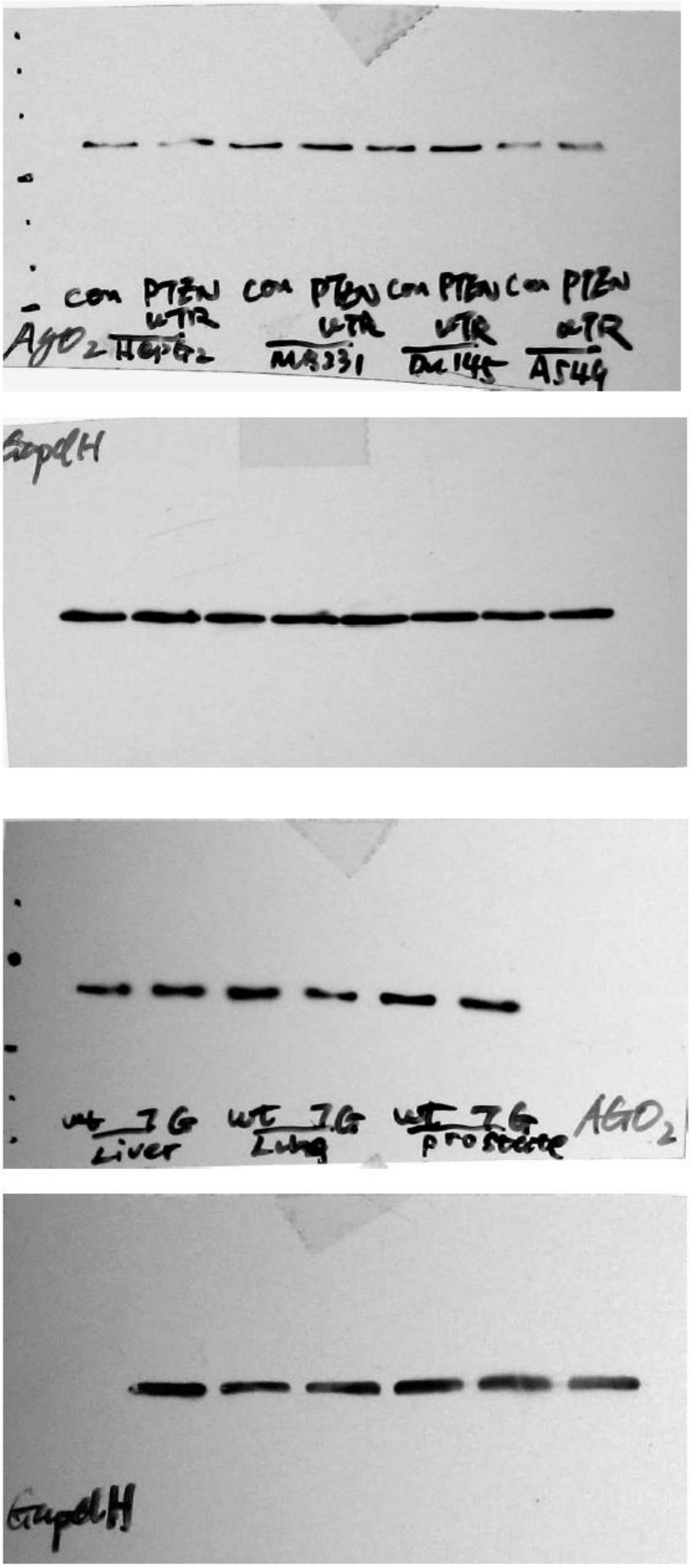

Figure S9. Full gel photos of Western blots.
